# Altered static and dynamic intrinsic brain activity in unilateral sudden sensorineural hearing loss

**DOI:** 10.3389/fnins.2023.1257729

**Published:** 2023-08-31

**Authors:** Jing Li, Xiaocheng Yu, Yan Zou, Yangming Leng, Fan Yang, Bo Liu, Wenliang Fan

**Affiliations:** ^1^Department of Radiology, Union Hospital, Tongji Medical College, Huazhong University of Science and Technology, Wuhan, China; ^2^Hubei Province Key Laboratory of Molecular Imaging, Wuhan, China; ^3^Department of Thyroid and Breast Surgery, Wuhan No. 1 Hospital, Wuhan, China; ^4^Department of Otorhinolaryngology Head and Neck Surgery, Union Hospital, Tongji Medical College, Huazhong University of Science and Technology, Wuhan, China

**Keywords:** unilateral sudden sensorineural hearing loss, resting state functional magnetic resonance imaging, static fractional amplitude of low-frequency fluctuation, temporal variability, intrinsic brain activity

## Abstract

**Introduction:**

Sudden sensorineural hearing loss (SSHL) is a critical otologic emergency characterized by a rapid decline of at least 30 dB across three consecutive frequencies in the pure-tone audiogram within a 72-hour period. This audiological condition has been associated with alterations in brain cortical and subcortical structures, as well as changes in brain functional activities involving multiple networks. However, the extent of cerebral intrinsic brain activity disruption in SSHL remains poorly understood. The aimed of this study is to investigate intrinsic brain activity alterations in SSHL using static and dynamic fractional amplitude of low-frequency fluctuation (fALFF) analysis.

**Methods:**

Resting-state functional magnetic resonance imaging (fMRI) data were acquired from a cohort of SSHL patients (unilateral, *n* = 102) and healthy controls (*n* = 73). Static and dynamic fALFF methods were employed to analyze the acquired fMRI data, enabling a comprehensive examination of intrinsic brain activity changes in SSHL.

**Results:**

Our analysis revealed significant differences in static fALFF patterns between SSHL patients and healthy controls. SSHL patients exhibited decreased fALFF in the left fusiform gyrus, left precentral gyrus, and right inferior frontal gyrus, alongside increased fALFF in the left inferior frontal gyrus, left superior frontal gyrus, and right middle temporal gyrus. Additionally, dynamic fALFF analysis demonstrated elevated fALFF in the right superior frontal gyrus and right middle frontal gyrus among SSHL patients. Intriguingly, we observed a positive correlation between static fALFF in the left fusiform gyrus and the duration of hearing loss, shedding light on potential temporal dynamics associated with intrinsic brain activity changes.

**Discussion:**

The observed disruptions in intrinsic brain activity and temporal dynamics among SSHL patients provide valuable insights into the functional reorganization and potential compensatory mechanisms linked to hearing loss. These findings underscore the importance of understanding the underlying neural alterations in SSHL, which could pave the way for the development of targeted interventions and rehabilitation strategies aimed at optimizing SSHL management.

## Introduction

Sudden sensorineural hearing loss (SSHL) is an otologic emergency and characterized by a rapid decline of at least 30 dB across three consecutive frequencies in the pure-tone audiogram within a 72-h period. It is usually unilateral, while bilateral involvement is observed in fewer than 5% of cases (Schreiber et al., 2010; Murray et al., 2022). Based on previous reports, the reported incidence of SSHL ranges from 5 to 27 per 100,000 individuals, and its annual incidence continues to rise (Alexander and Harris, 2013; Kim et al., 2017; Kuo et al., 2019). Prior studies have suggested various potential causes of SSHL, such as vascular disorders, viral or bacterial infections, inner ear membrane rupture, tumor, and autoimmune diseases (Cadoni et al., 2002; Schreiber et al., 2010; Kim et al., 2017; Chandrasekhar et al., 2019). The current treatment modalities for SSHL include intratympanic corticosteroid injections (Filipo et al., 2013; Lavigne et al., 2016; Li and Ding, 2020; Plontke et al., 2022), hyperbaric oxygen therapy (Attanasio et al., 2015; Lamm et al., 2016), and oral corticosteroids (Swachia et al., 2016). However, despite the administration of suitable high-quality therapy, patients with SSHL often experience limited improvement in their hearing function, leading to potential adverse impacts on economic indicators for society and resulting in diminished long-term quality of life (Kuhn et al., 2011). Nonetheless, the etiology of over two-thirds of SSHL cases remains uncertain and is classified as idiopathic. What is worse, SSHL not only impacts the structure and function of the inner ear, including cochlear hair cells, auditory nerve fibers, and membranous labyrinth, but it also gives rise to central system symptoms such as cognitive deficits, depression, and anxiety, which may significantly diminish the quality of life for affected individuals (Schreiber et al., 2010; Chandrasekhar et al., 2019). Hence, the evaluation of comprehensive brain functions may aid in uncovering the underlying mechanisms of SSHL.

The advancement of imaging technology has revealed that individuals with SSHL exhibit not only alterations in brain cortical and subcortical structures (Yang et al., 2014; Fan et al., 2015; Qu et al., 2020) but also associated with changes in brain functional activities involving various brain networks such as the default mode, auditory, executive control, and visual networks (Xu et al., 2016; Chen et al., 2020; Minosse et al., 2021; Wang et al., 2021; Hua et al., 2022). The investigation of brain functional activity has become increasingly important in understanding the underlying neural mechanisms associated with SSHL. Amplitude of Low-Frequency Fluctuations (ALFF) is a widely used measure that reflects the intensity of spontaneous neural activity within specific brain regions. Several studies have utilized ALFF to investigate alterations in spontaneous brain activity in healthy control (Newbold et al., 2020; Elias et al., 2022) and various neurological and psychiatric disorders, such as Alzheimer’s disease (Liu et al., 2014), major depressive disorder (Gray et al., 2020), schizophrenia (Sui et al., 2018) and traumatic brain injury (Palacios et al., 2013). These studies have demonstrated the utility of ALFF in providing valuable insights into the functional changes that occur in the brain across different conditions. However, these studies have primarily focused on assessing the “static” temporal strength or inter-region relationship in the temporal pattern, thereby overlooking the investigation of dynamic brain alterations or the time-varying nature of the blood oxygen level dependent signal in resting-state functional MRI scanning. Measuring temporal variability offers valuable insights into the fluctuation patterns of brain activity amplitudes over time (Leonardi and Van De Ville, 2015; Yan et al., 2017).

Recent studies have increasingly proposed that the dynamic ALFF or dynamic fractional ALFF (fALFF) provides an approach to measure the alterations of temporal variability of intrinsic neural activity in many diseases (Wang et al., 2019; Ma et al., 2020a; Zhou et al., 2021; Dong et al., 2022). ALFF measures the amplitude of low-frequency fluctuations in the BOLD signal and provides information about the absolute intensity of neural activity within specific frequency bands, typically the low-frequency range (0.01–0.1 Hz). And ALFF has been extensively used to investigate regional spontaneous activity and has been associated with the baseline functional state of the brain. On the other hand, fALFF calculates the ratio of the power of low-frequency fluctuations to the total power across the entire frequency range, providing a measure of the relative contribution of low-frequency oscillations to the global brain signal. This normalization process is particularly useful in reducing the influence of physiological noise, making fALFF more robust and less sensitive to non-neural fluctuations (Zou et al., 2008). While ALFF provides localized information about absolute neural activity, fALFF offers a more global perspective on the brain’s intrinsic activity.

By employing dynamic ALFF analysis, it has been observed that individuals with SSHL demonstrate decreased dynamic ALFF variability specifically in the bilateral inferior occipital gyrus, middle occipital gyrus, calcarine, right lingual gyrus, and right fusiform gyrus (Li et al., 2022). These findings indicate that SSHL patients may undergo cross-modal plasticity and visual compensation, which could be closely linked to the underlying pathophysiology of SSHL (Li et al., 2022). Therefore, studying these dynamic characteristics can potentially reveal additional pathological changes associated with SSHL and provide valuable guidance for more effective treatment strategies. Given the mentioned limitation of small sample size mentioned by the authors and the inherent heterogeneity of SSHL, conducting studies on dynamic intrinsic brain activity in SSHL with a larger sample size is indeed necessary, which would enhance the generalizability and statistical power of the findings, allowing for more robust conclusions to be drawn regarding the dynamic characteristics of intrinsic brain activity in individuals with SSHL.

In the current work, we utilized the fALFF combined with a sliding window approach to evaluate the temporal variability of intrinsic brain activity in individuals with SSHL. We hypothesized that SSHL patients would exhibit altered patterns of static and dynamic fALFF in comparison to healthy controls (HCs). By identifying regions with altered fALFF values, it may provide valuable biomarkers associated with SSHL. These biomarkers could serve as objective and quantifiable indicators of neural dysfunction in SSHL patients, aiding in early diagnosis and individualized treatment strategies. Moreover, we hypothesized that dynamic fALFF could reveal underlying abnormal intrinsic brain activity that static ALFF measures might not capture, thereby contributing to a deeper understanding of the physiological mechanisms underlying SSHL. It is hoped these results could offer valuable insights into the neural circuits and pathways involved in the pathophysiology of the SSHL. Additionally, we proposed that the dynamic indexes might be associated with clinical characteristics of SSHL. The identification of specific fALFF patterns associated with the severity and progression of SSHL may offer prognostic information for individual patients. By correlating fALFF findings with clinical outcomes, we can potentially identify patients at higher risk of developing complications or poorer outcomes, facilitating early intervention and personalized care.

## Materials and methods

### Subjects

A total of 175 subjects, which consisted of 102 participants with unilateral SSHL and 73 sex- and age-matched HC subjects, were recruited from Wuhan Union Hospital. All participants were right-handed. Demographic and clinical data for the subjects are shown in Table 1. No significant differences were found in gender, age, education level between SSHL patients and HC subjects. Pure-tone audiometry was performed using seven different octave frequencies (0.125, 0.25, 0.5, 1, 2, 4, and 8 kHz) to measure the pure tone average (PTA) and determine the hearing level. As described in our previous study (Leng et al., 2022), PTA was calculated as the simple arithmetic mean for these seven frequencies. The initial pure-tone audiometry curves were categorized into four types: (1) low-frequency hearing loss (the average threshold of 0.25 and 0.5 kHz is 20 dB higher than that of 4 and 8 kHz), (2) high-frequency hearing loss (the average threshold of 4 and 8 kHz is 20 dB higher than that of 0.25 and 0.5 kHz), (3) flat-type hearing loss (similar threshold is observed across the entire frequency range and the average threshold is less than 90 dB HL), and (4) profound hearing loss (the average threshold of 0.5, 1, 2, and 4 kHz exceeds 90 dB HL) (Leng et al., 2022).

**Table 1:** Demographic and clinical variables.

Subjects	SSHL	HC	*p* value
Number of subjects	102	73	N/A
Age (years)	38.89 ± 12.11	38.01 ± 16.52	0.685
Gender (male/female)	52/50	37/36	0.969
Education level (years)	12.67 ± 3.20	12.74 ± 2.93	0.878
Duration (days)	9.03 ± 4.27	N/A	N/A
Effected side (left/right)	56/46	N/A	N/A
PTA (dB)	73.66 ± 10.38	13.25 ± 5.08	<0.001
THI score	47.54 ± 26.37	N/A	N/A

A Pearson chi-squared test was used for gender comparison. Independent samples *t*-tests were used for age, education and PTA comparisons.

SSHL, sudden sensorineural hearing loss; HC, healthy control; PTA, pure tone average; THI, tinnitus handicap inventory.

The diagnosis of SSHL was established following the diagnostic guidelines of SSHL outlined by the Clinical Practice Guideline (Chandrasekhar et al., 2019). Exclusion criteria were as follows: (1) SSHL patients with bilateral hearing loss, (2) all subjects had neurological diseases (such as acoustic neuroma, brain tumors, and trauma), psychiatric diseases (such as depression and insomnia), and other otolaryngological diseases (such as otitis media, Meniere’s disease, and pulsatile tinnitus), and (2) all subjects had claustrophobia and cardiac pacemakers. Due to the frequent occurrence of tinnitus and/or vertigo in SSHL, the severity of tinnitus was assessed using the Tinnitus Handicap Inventory (THI). The THI utilizes a scale ranging from 0 to 100 to measure the increasing level of handicap caused by tinnitus (Newman et al., 1996).

This study was approved by the Tongji Medical College of Huazhong University of Science and Technology medical ethics committee. All subjects were informed about the purpose of the study before giving their written consents in accordance with Chinese legislation.

### Data acquisition

The study employed a 3 T MRI system (Siemens Trio Tim, Erlangen, Germany) with a 12-channel head coil to acquire anatomical and functional images. Prior to administering any drug treatment, all patients underwent imaging experiments during which they were instructed to remain still and with their eyes closed while wearing headphones to minimize noise. A foam cushion was used to reduce head movements and motion artifacts. Anatomical images were obtained using a 3-dimensional high-resolution T1-weighted magnetization-prepared rapid acquisition gradient echo (MP-RAGE) sequence with the following parameters: repetition time (TR) = 2,250 ms, echo time (TE) = 2.26 ms, inversion time (TI) = 900 ms, flip-angle = 9°, voxel size = 1.0 × 1.0 × 1.0 mm^3^, field of view (FOV) = 256 mm × 256 mm, slice thickness = 1.00 mm, and 176 sagittal slices covering the entire brain. Functional images were acquired using a gradient echo type echo planar imaging (EPI) sequence with the following parameters: TR = 2000 ms, TE = 30 ms, flip-angle = 90°, voxel size = 3.0 × 3.0 × 3.0 mm^3^, and FOV = 200 mm × 200 mm, resulting in 240 images. Additionally, a T2-weighted sequence was acquired to assess the status of the peripheral auditory system using the following parameters: TR = 1,000 ms, TE = 132 ms, slice thickness = 0.5 mm, slice number = 64, flip-angle = 120°, FOV = 200 mm × 200 mm, and averages = 2. Two radiologists independently reviewed the MR images for abnormalities such as otitis media, acoustic neuroma, and brain tumors, and any subjects with such abnormalities were excluded from subsequent analysis.

### Data preprocessing

The Brain Imaging Data Processing and Analysis (DPABI, v7.0) tool (Yan et al., 2016), based on the Statistical Parametric Mapping (SPM12) toolkits (https://www.fil.ion.ucl.ac.uk/spm/software/spm12/), was used to preprocess the resting-state BOLD data (Yan et al., 2013). The first 10 volumes were discarded to eliminate T1 equilibration effects, resulting in 230 time points. Slice timing correction and realignment correction were then performed to address different slice acquisition times and head motion using six-parameter rigid-body transformation. Nuisance covariates, including linear trends, the white matter signal, cerebrospinal fluid signal, and Friston 24 head motion parameters, were regressed out from the functional signal. Normalization was carried out by coregistering functional images with corresponding structural images, which were segmented and normalized to the standard Montreal Neurological Institute (MNI) template using the Diffeomorphic Anatomical Registration Through Exponentiated Lie algebra (DARTEL) tool. Participants with maximum head motion larger than 1.5 mm in displacement or 1.5° in rotation, and mean frame-wise displacement (FD) calculated by Jenkinson method larger than 0.2 were excluded from the analysis.

### Static and dynamic fALFF calculation

After normalization, the time series of functional imaging underwent a fast Fourier transform to transform them into the frequency domain. The power spectrum and square root transformation were then used to calculate the ALFF. The ALFF in the low frequency band (0.01–0.1 Hz) was divided by the amplitude in the entire frequency band to obtain the fALFF (Zou et al., 2008). The fALFF data were then transformed into z maps using Fisher’s r-to-z transformation to enhance normality for group-level analysis. Finally, a 6 mm full width at half-maximum (FWHM) Gaussian kernel was applied to smooth the functional images.

A sliding window approach via DPABI was used to compute the dynamic fALFF (Yan et al., 2017). The window length is a critical parameter when computing resting-state dynamics. Using a shorter window length can heighten the risk of introducing spurious fluctuations in the observed dynamic fALFF. On the other hand, opting for a longer window length may impede the accurate depiction of the temporal variability dynamics of fALFF (Leonardi and Van De Ville, 2015; Li et al., 2019). To avoid spurious fluctuations caused by a window length shorter than the minimum frequency of the time series (fmin), the window length was set to 50 TRs (100 s), which was longer than 1/fmin (Lawrence and Thevasagayam, 2015; Yan et al., 2017; Li et al., 2019). The window was shifted by 5 TRs (step size: 10 s) to capture temporal variability in fALFF. This approach generated 37 windows for each participant. The mean and standard deviation of each voxel were computed in each sliding window to assess variability in dynamic fALFF, which was expressed as the coefficient of variation (CV: standard deviation/mean). The CV maps were then Z-standardized and smoothed with a 6 mm full width at half-maximum (FWHM) Gaussian kernel for further statistical analysis (Zhou et al., 2021).

### Statistical analysis

To investigate the spatial distribution, group means of static and dynamic fALFF were computed. Following that, a two-sample t-test analysis was conducted to assess the differences in static and dynamic fALFF between the SSHL and HC groups. Voxel-level significance was set at *p* < 0.001, with cluster-level correction using Gaussian random field (GRF) at *p* < 0.01. Age, sex, mean FD, education, and gray matter volume were included as covariates in the analysis. Furthermore, partial correlation analyses were conducted to examine the association between the altered functional index of static or dynamic fALFF and clinical assessments, with sex, age, mean FD, education, and gray matter volume included as covariates (Zhou et al., 2021). Besides, we also conducted a subgroup analysis to explore potential differences in fALFF patterns between patients with left-sided SSNHL and those with right-sided SSNHL, and the results were showed in Supplementary material (see Supplementary Figures S1-S5; Supplementary Table S1).

## Results

### Spatial distribution and group differences of static fALFF

**Figure 1:**
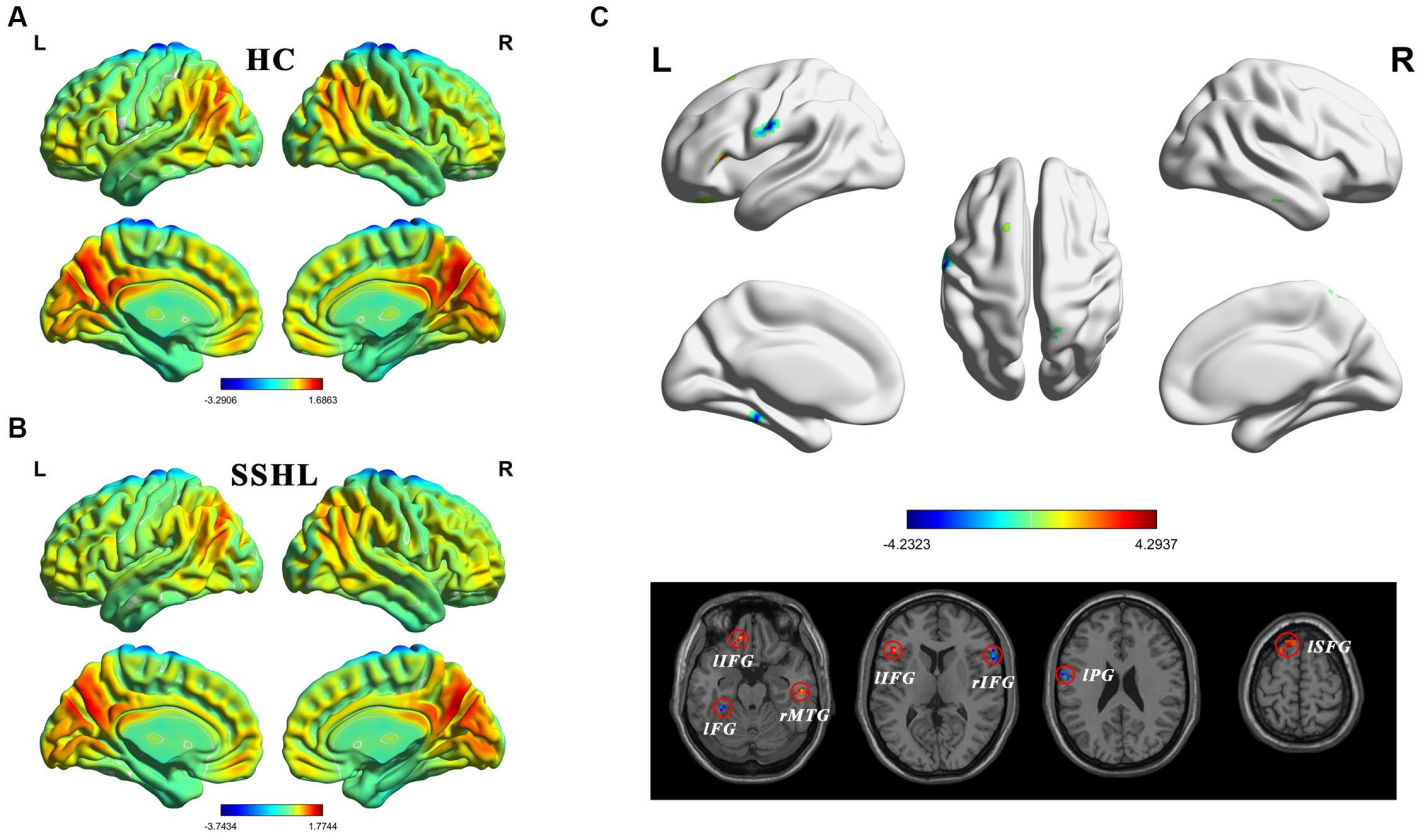
Altered static intrinsic brain activity in unilateral SSHL. **(A)** Within group mean static fALFF maps in the HC. **(B)** Within group mean static fALFF maps in the SSHL. **(C)** Group comparisons of static fALFF between the SSHL and HC groups (Gaussian random field correction; voxel-level *p* < 0.001, cluster-level *p* < 0.01). SSHL, sudden sensorineural hearing loss; HC, healthy controls; L, left; R, right; lFG, left fusiform gryus; lPG, left precentral gyrus; rIFG, right inferior frontal gyrus; lIFG, left inferior frontal gyrus; lSFG, left superior frontal gyrus; rMTG, right middle temporal gyrus.

Figure 1 shows the spatial distribution of static fALFF (Figures 1A,B) in the SSHL and HC groups. Compared with the HCs, the SSHL patients exhibited significantly (GRF correction; voxel-level *p* < 0.001, cluster-level *p* < 0.01) decreased static fALFF in the left fusiform gryus (lFG), left precentral gyrus (lPG) and right inferior frontal gyrus (rIFG), and increased static fALFF in the left inferior frontal gyrus (lIFG), left superior frontal gyrus (lSFG) and right middle temporal gyrus (rMTG) (Figure 1C; Table 2).

**Table 2:** Comparison of static and dynamic fALFF between the SSHL and HC Groups (voxel-level *p* < 0.001 and GRF corrected at cluster-level *p* < 0.01).

Brain Regions	Voxels	MNI coordinates			*T* values
		X Y Z			
Static fALFF (SSHL vs. HC)					
Left fusiform gyrus (lFG)	37	−30	−36	−33	−4.6
Left inferior frontal gyrus (lIFG)	47	−12	39	−21	4.2
Right middle temporal gyrus (rMTG)	21	51	−24	−18	4.6
Left inferior frontal gyrus (lIFG)	45	−45	24	9	3.8
Right inferior frontal gyrus (rIFG)	44	60	18	12	−4.1
Left precentral gyrus (lPG)	14	−57	−9	27	−4.4
Left superior frontal gyrus (lSFG)	16	−21	18	63	3.7
Dynamic fALFF (SSHL vs. HC)					
Right superior frontal gyrus (rSFG)	14	18	45	30	4.0
Right middle frontal gyrus (rMFG)	16	15	9	63	3.8

MNI, montreal neurological institute; SSHL, sudden sensorineural hearing loss; HC, healthy control.

### Spatial distribution and group differences of dynamic fALFF

**Figure 2:**
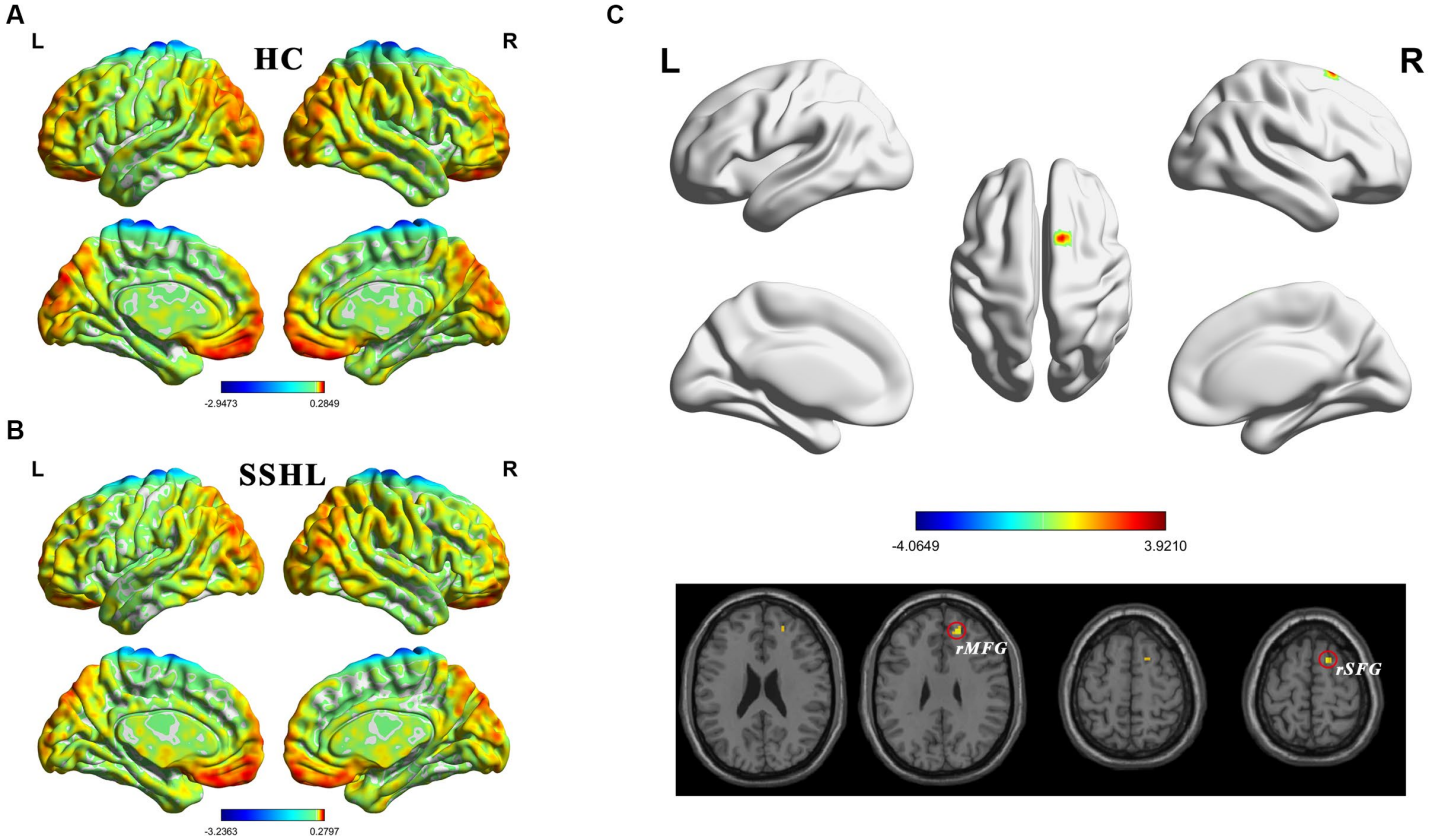
Altered dynamic intrinsic brain activity in unilateral SSHL. **(A)** Within group mean dynamic fALFF maps in the HC. **(B)** Within group mean dynamic fALFF maps in the SSHL. **(C)** Group comparisons of dynamic fALFF between the SSHL and HC groups (Gaussian random field correction; voxel-level *p* < 0.001, cluster-level *p* < 0.01). SSHL, sudden sensorineural hearing loss; HC, healthy controls; L, left; R, right; rSFG, right superior frontal gyrus; rMFG, right middle frontal gyrus.

Figure 2 shows the spatial distribution of dynamic fALFF (Figures 2A,B) in the SSHL and HC groups. Compared with the HCs, the SSHL patients only exhibited significantly (GRF correction; voxel-level *p* < 0.001, cluster-level *p* < 0.01) increased dynamic fALFF in the right superior frontal gyrus (rSFG) and right middle frontal gyrus (rMFG) (Figure 2C; Table 2).

### Correlation analysis

**Figure 3:**
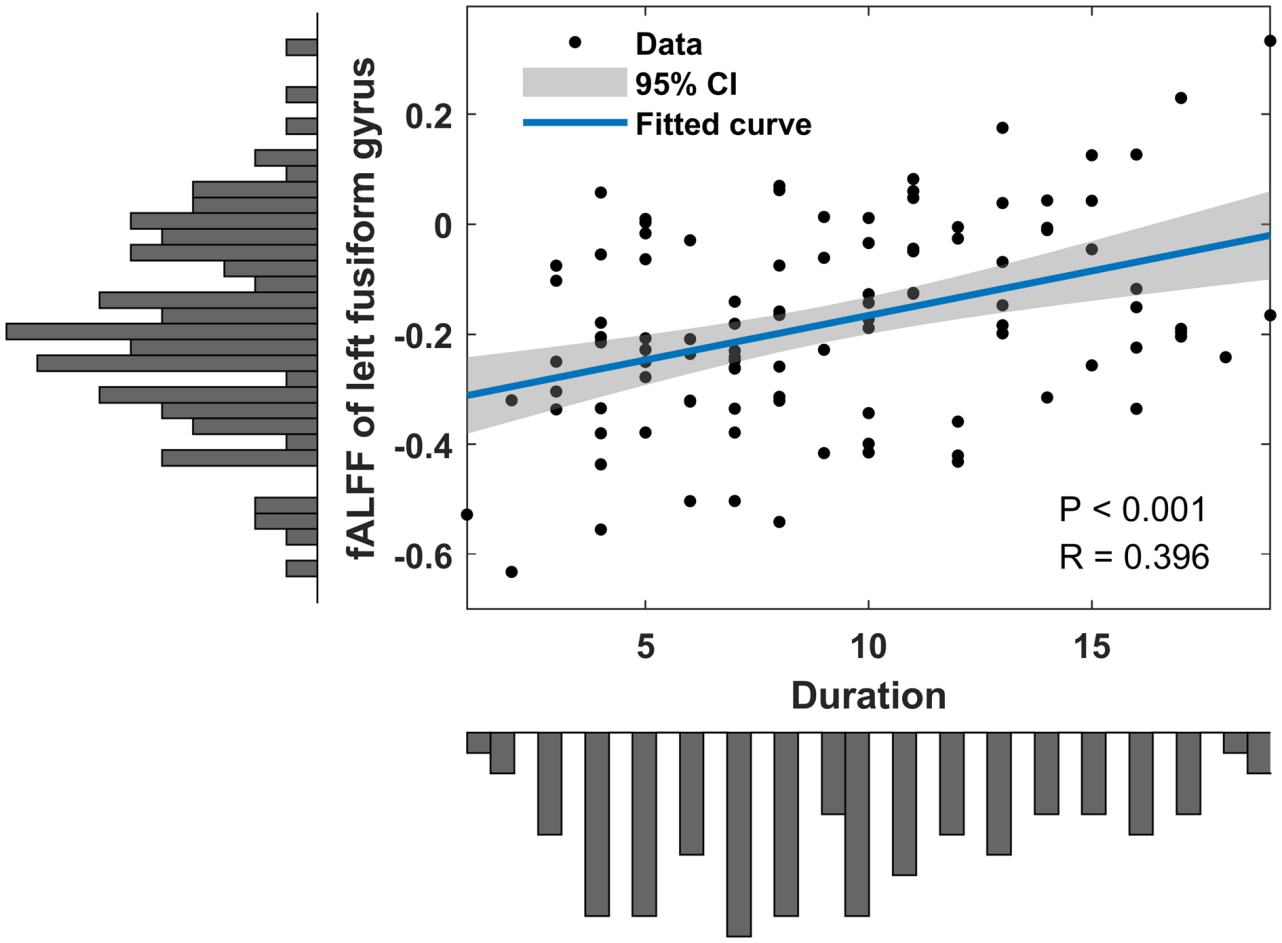
Clinical associations of altered static fALFF in SSHL patients. SSHL, sudden sensorineural hearing loss; fALFF, fractional amplitude of low-frequency fluctuation.

Correlation analyses identified positive correlations between static fALFF in left fusiform gyrus and hearing loss duration (*R* = 0.396, *p* < 0.001) (Figure 3). No significant correlation was found between dynamic fALFF and clinical measurements.

## Discussion

The results of the study revealed significant intrinsic brain activity differences in static and dynamic fALFF patterns in various brain regions between individuals with SSHL and HCs. Moreover, static fALFF in left fusiform gyrus was positively correlated with hearing loss duration. Our findings revealed static and dynamic changes of brain functional intrinsic activities in SSHL patients which provide the neurophysiological basis for SSHL.

In terms of static fALFF, we found SSHL patients exhibited significantly decreased static fALFF in the left fusiform gryus, left precentral gyrus and right inferior frontal gyrus, which suggests reduced spontaneous neural activity in these regions. The fusiform gyrus is known to be involved in high-order visual information processing, including face recognition and object perception (Weiner and Zilles, 2016; Palejwala et al., 2020). It is reported that unilateral hearing loss showed reduced grey matter volumes in fusiform gyrus (Heggdal et al., 2020). Indeed, alterations in the brain activity of the fusiform gyrus have been reported in various neurological conditions, including amyotrophic lateral sclerosis (Luo et al., 2012), Parkinson’s disease (Pan et al., 2017), obsessive–compulsive personality disorder (Lei et al., 2020) and social anxiety disorder (Qiu et al., 2015). The precentral gyrus is a key region in the primary motor cortex, responsible for voluntary motor control (Silva et al., 2022; Wei et al., 2022). The inferior frontal gyrus has been implicated in various cognitive processes, including language production and executive functions (Hampshire et al., 2010; Ishkhanyan et al., 2020). Previous studies have reported similar intrinsic brain activity alterations in hearing loss patients (Zhou et al., 2019; Chen et al., 2020; Yang et al., 2021). For instance, Chen et al., (2020) found decreased static ALFF and fALFF in the auditory cortex and visual cortex in unilateral SSHL, and Zhou et al. (2019) found abnormal static ALFF in the inferior frontal gyrus in unilateral acute tinnitus patients with hearing loss. The observed decrease in static fALFF in these regions may reflect alterations in sensory and cognitive functions associated with SSHL.

Conversely, the increased static fALFF in the left inferior frontal gyrus, left superior frontal gyrus, and right middle temporal gyrus suggests enhanced neural activity in these areas. The superior frontal gyrus is implicated in cognitive control, working memory, and attentional processes (du Boisgueheneuc et al., 2006; Briggs et al., 2020). Indeed, alterations in the intrinsic brain activity of the superior frontal gyrus have been reported in various conditions, including single-sided deafness (Qiao et al., 2022), Alzheimer’s disease with depression (Mu et al., 2020) and left-sided long-term hearing impairment (Xie et al., 2019). The middle temporal gyrus is involved in auditory processing and language comprehension (Yu et al., 2022; Sugimoto et al., 2023). The increased fALFF in the superior frontal gyrus observed in individuals with SSHL is consistent with previous findings reported in the literature (Chen et al., 2020). The increased static fALFF in these regions may indicate compensatory mechanisms or heightened cognitive engagement in response to the hearing loss experienced in SSHL. The observed differences in static fALFF patterns suggest that individuals with SSHL may exhibit both hypoactive and hyperactive neural responses compared to HCs (Liu et al., 2021; Yao et al., 2021). These alterations could be associated with compensatory mechanisms or functional changes in response to the hearing loss.

Interestingly, when examining dynamic fALFF, SSHL patients only displayed significantly increased dynamic fALFF in the right superior frontal gyrus and right middle frontal gyrus compared to HCs. The middle frontal gyrus plays a role in working memory and attention (Briggs et al., 2021; Martin-Luengo et al., 2023). Static fALFF represents the fractional amplitude of low-frequency fluctuations calculated over the entire resting-state fMRI scan duration, providing a snapshot of regional spontaneous brain activity. On the other hand, dynamic fALFF captures the temporal variability of fALFF values throughout the entire resting-state scan, offering insights into how brain activity dynamically changes over time. These findings indicate that SSHL patients exhibit altered temporal variability in neural activity specifically within these regions. Increased dynamic fALFF in these regions has also been reported in individuals with Alzheimer’s disease (Li et al., 2021), major depressive disorder with suicidal ideation (Yang et al., 2022), and amyotrophic lateral sclerosis (Ma et al., 2020b). These results suggest that dynamic fALFF analysis provides unique insights into the time-varying nature of intrinsic brain activity in SSHL. These dynamic alterations may reflect adaptive or compensatory mechanisms to cope with the hearing loss or could be related to the underlying pathophysiology of SSHL.

These findings collectively highlight the involvement of various brain regions in SSHL, indicating potential disruptions in sensory, cognitive, and attentional processes. The alterations in both static and dynamic fALFF provide valuable insights into the complex neural changes associated with SSHL, contributing to a deeper understanding of the condition. Overall, the study highlights the presence of both static and dynamic fALFF alterations in SSHL patients compared to HCs. These measures provide complementary information, allowing us to gain insights into both stable alterations in brain activity and dynamic fluctuations that may not be apparent in static measures alone. The findings suggest that SSHL is associated with disrupted intrinsic brain activity in multiple regions, involving both increased and decreased neural activity. The differential patterns observed in static and dynamic fALFF emphasize the importance of investigating both static and dynamic aspects of intrinsic brain activity to gain a more comprehensive understanding of the neural changes associated with SSHL.

Additionally, in our study, we observed positive correlations between static fALFF in the left fusiform gyrus and the duration of hearing loss in individuals with SSHL. This finding suggests that the severity or duration of hearing loss may be associated with altered neural activity in the fusiform gyrus, which implies a potential compensatory mechanism or adaptive changes in auditory processing associated with prolonged hearing loss. It is important to note that the positive correlation observed in our study does not imply a causative relationship, and further research is needed to elucidate the underlying mechanisms. Nevertheless, these findings provide preliminary evidence of the potential link between the duration of hearing loss and altered neural activity in the fusiform gyrus.

Despite the significant findings of our study, several limitations should be acknowledged. Firstly, the cross-sectional design of the study prevented us from establishing causal relationships between the observed brain activity alterations and SSHL symptom duration. Longitudinal studies are warranted to elucidate the temporal dynamics and progression of these changes over time. It would be essential to explore changes in brain activity over multiple time points during the course of SSHL to better understand their persistence or potential resolution over time. Secondly, our study focused specifically on investigating fALFF as an indicator of intrinsic brain activity alterations in SSHL. While fALFF is a valuable metric, we acknowledge that other functional connectivity metrics or brain imaging modalities, such as seed-based functional connectivity analysis or diffusion tensor imaging, could provide additional insights into the neural changes associated with SSHL. Further studies incorporating a broader range of imaging modalities may offer a more comprehensive understanding of the underlying pathophysiological mechanisms of SSHL. Thirdly, it is important to acknowledge that all patients included in our study exhibited unilateral SSHL. As SSHL is typically unilateral, with bilateral involvement accounting for less than 5% of the cases, we were unable to perform a subgroup analysis based on the bilateral side of SSHL. In future studies, if a sufficient number of bilateral SSHL cases are available, a specific analysis to investigate these aspects may gain deeper insights into the neural mechanisms associated with bilateral SSHL. Finally, the heterogeneity of SSHL, including variations in the etiology, duration, and severity of hearing loss, should be considered when interpreting the results. Future studies should consider these factors and incorporate more comprehensive assessments to further elucidate the complex mechanisms underlying SSHL.

## Conclusion

In conclusion, our study on SSHL revealed significant alterations in both static and dynamic fractional amplitude of low-frequency fluctuation (fALFF) patterns, providing valuable insights into the intrinsic brain activity changes associated with the condition. SSHL patients exhibited decreased static fALFF in the left fusiform gyrus, left precentral gyrus, and right inferior frontal gyrus, indicating reduced spontaneous neural activity, while increased static fALFF was observed in the left inferior frontal gyrus, left superior frontal gyrus, and right middle temporal gyrus, suggesting heightened neural activity and potential compensatory mechanisms. Additionally, SSHL patients showed increased dynamic fALFF in the right superior frontal gyrus and right middle frontal gyrus, indicating altered temporal variability of neural activity within these frontal regions. Furthermore, a positive correlation was found between static fALFF in the left fusiform gyrus and the duration of hearing loss in SSHL, suggesting a potential association between hearing loss severity and altered neural activity in this region. These findings emphasize the complex nature of intrinsic brain activity changes in SSHL and provide insights into the functional reorganization and compensatory mechanisms that occur in response to hearing loss. Further research is needed to explore the functional significance of these alterations, develop targeted interventions, and optimize rehabilitation approaches for SSHL management.

## Data availability statement

The raw data supporting the conclusions of this article will be made available by the authors, without undue reservation.

## Ethics statement

The studies involving humans were approved by the Tongji Medical College of Huazhong University of Science and Technology medical ethics committee. The studies were conducted in accordance with the local legislation and institutional requirements. The participants provided their written informed consent to participate in this study.

## Author contributions

JL: conceptualization, data curation, formal analysis, methodology, visualization, writing – original draft. XY: methodology, data curation, formal analysis, software, validation, writing – original draft. YZ: data curation, investigation, methodology. YL: data curation, funding acquisition, resources, validation, writing – review and editing. FY: resources, supervision, validation, writing – review and editing. BL: conceptualization, funding acquisition, investigation, methodology, supervision, writing – review and editing. WF: conceptualization, funding acquisition, investigation, methodology, project administration.

## Funding

The author(s) declare financial support was received for the research, authorship, and/or publication of this article.

This research was supported by the National Natural Science Foundation of China (grant no. 82101231, 81701673 and 81670930), and Natural Science Foundation of Hubei Province, China (no. 2021CFB547 and 2019CFB497).

## Conflict of interest

The authors declare that the research was conducted in the absence of any commercial or financial relationships that could be construed as a potential conflict of interest.

## Publisher’s note

All claims expressed in this article are solely those of the authors and do not necessarily represent those of their affiliated organizations, or those of the publisher, the editors and the reviewers. Any product that may be evaluated in this article, or claim that may be made by its manufacturer, is not guaranteed or endorsed by the publisher.
