# Supplementary material for: Altered static and dynamic intrinsic brain activity in unilateral sudden sensorineural hearing loss
Source: Front Neurosci. 2023 Aug 31;17:1257729. doi: 10.3389/fnins.2023.1257729 (PMC10500124; doi:10.3389/fnins.2023.1257729)
Supplement: Supplementary file 1 [file Data_Sheet_1.pdf]

Spatial distribution and group differences of static fALFF

Compared with the HCs, the left side SSHL patients exhibited significantly (GRF correction; voxel-level $p < 0.001$, cluster-level $p < 0.01$) decreased static fALFF in the left postcentral gyrus(lPosG), right postcentral gyrus (rPosG) and left precentral gyrus(lPreG), and increased static fALFF in the left inferior frontal gyrus(lIFG), right middle temporal gyrus(rMTG), left superior frontal gyrus(lSFG) and right supramarginal gyrus(rSG) (Figure S1, Table S1). While the right side SSHL patients exhibited significantly (GRF correction; voxel-level $p < 0.001$, cluster-level $p < 0.01$) increased static fALFF in the left orbital gyrus(lOG) and left middle frontal gyrus(lMFG) (Figure S2, Table S1). Moreover, the left side SSHL patients exhibited significantly (GRF correction; voxel-level $p < 0.001$, cluster-level $p < 0.01$) decreased static fALFF in the right superior temporal gyrus(rSTG) when compared to right side SSHL patients (Figure S3, Table S1).

Table S1

Comparison of static and dynamic fALFF between the SSHL and HC Groups (voxel-level $p < 0.001$ and GRF corrected at cluster-level $p < 0.01$)

Brain Regions	Voxels	MNI Coordinates			*T* Values
		X	Y	Z	
Static fALFF (left side SSHL vs. HC)					

Left inferior frontal gyrus(lIFG)	15	-12	39	-21	3.9
Right middle temporal gyrus(rMTG)	28	54	-18	-21	4.7
Left postcentral gyrus(lPosG)	17	-45	-36	51	-3.9
Right postcentral gyrus (rPosG)	44	60	18	12	-4.1
Left precentral gyrus(lPreG)	14	-60	-18	39	-4.3
Left superior frontal gyrus(lSFG)	88	-9	30	63	4.1
Right supramarginal gyrus(rSG)	23	42	-45	33	3.6
Static fALFF (right side SSHL vs. HC)					
Left orbital gyrus(lOG)	34	-3	33	-30	3.8
Left middle frontal gyrus(lMFG)	37	-30	48	-9	4.6
Static fALFF (left side SSHL vs. right side SSHL)					
Right superior temporal gyrus(rSTG)	25	60	-54	18	-3.8
Dynamic fALFF (left side SSHL vs. HC)					
Right cuneus	14	12	-93	15	4.3
Right superior parietal	16	27	-63	69	-3.7
Right superior frontal gyrus(rSFG)	17	0	33	63	3.3
Dynamic fALFF (left side SSHL vs. right side SSHL)					

Left precuneus	11	-6	-72	57	-3.6

MNI, montreal neurological institute; SSHL, sudden sensorineural hearing loss; HC, healthy control.

Spatial distribution and group differences of dynamic fALFF

Compared with the HCs, the left side SSHL patients exhibited significantly (GRF correction; voxel-level $p < 0.001$, cluster-level $p < 0.01$) increased dynamic fALFF in the right cuneus and right superior frontal gyrus(rSFG), and decreased dynamic fALFF in the right superior parietal (Figure S4, Table S1). However, there is no significant dynamic fALFF difference between right side SSHL patients and HCs. The left side SSHL patients exhibited significantly (GRF correction; voxel-level $p < 0.001$, cluster-level $p < 0.01$) decreased dynamic fALFF in the left precuneus when compared to right side SSHL patients (Figure S5, Table S1).

Figure and captions：

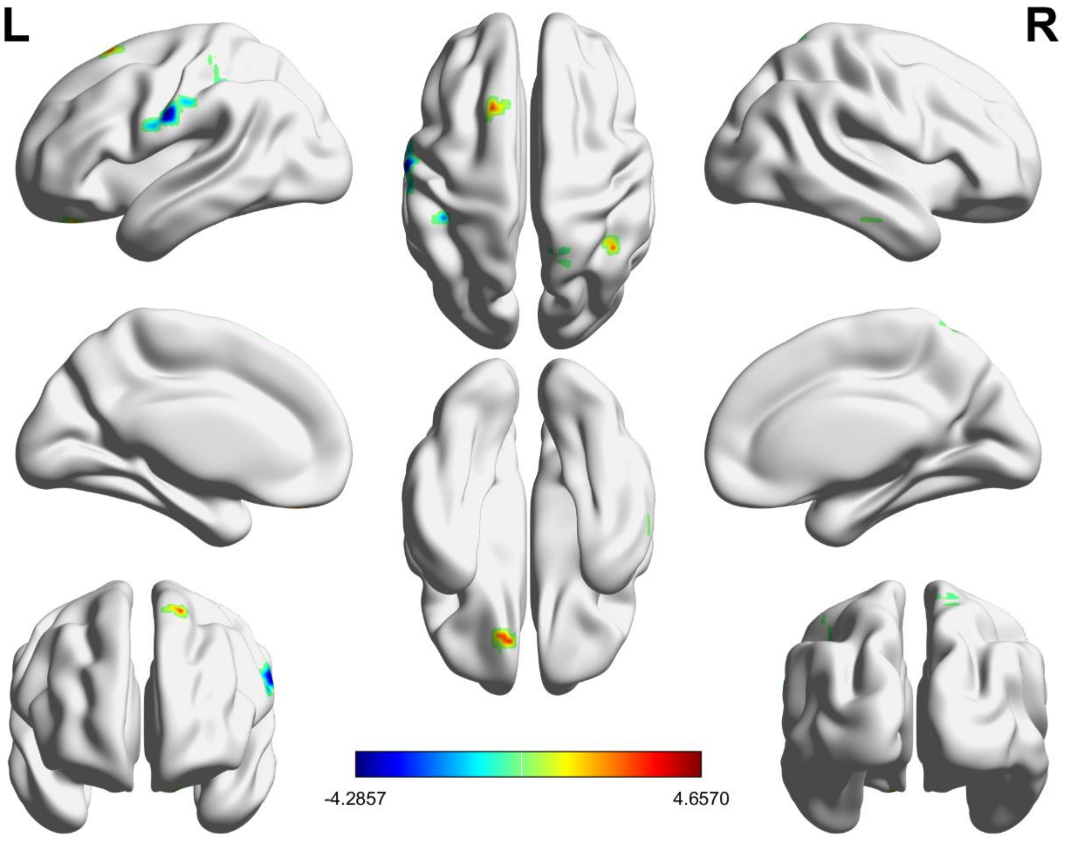

Figure S1.

Group comparisons of static fALFF between the left side SSHL and HC groups

(Gaussian random field correction; voxel-level $p < 0.001$, cluster-level $p < 0.01$). SSHL, sudden sensorineural hearing loss; HC, healthy controls; L, left; R, righ.

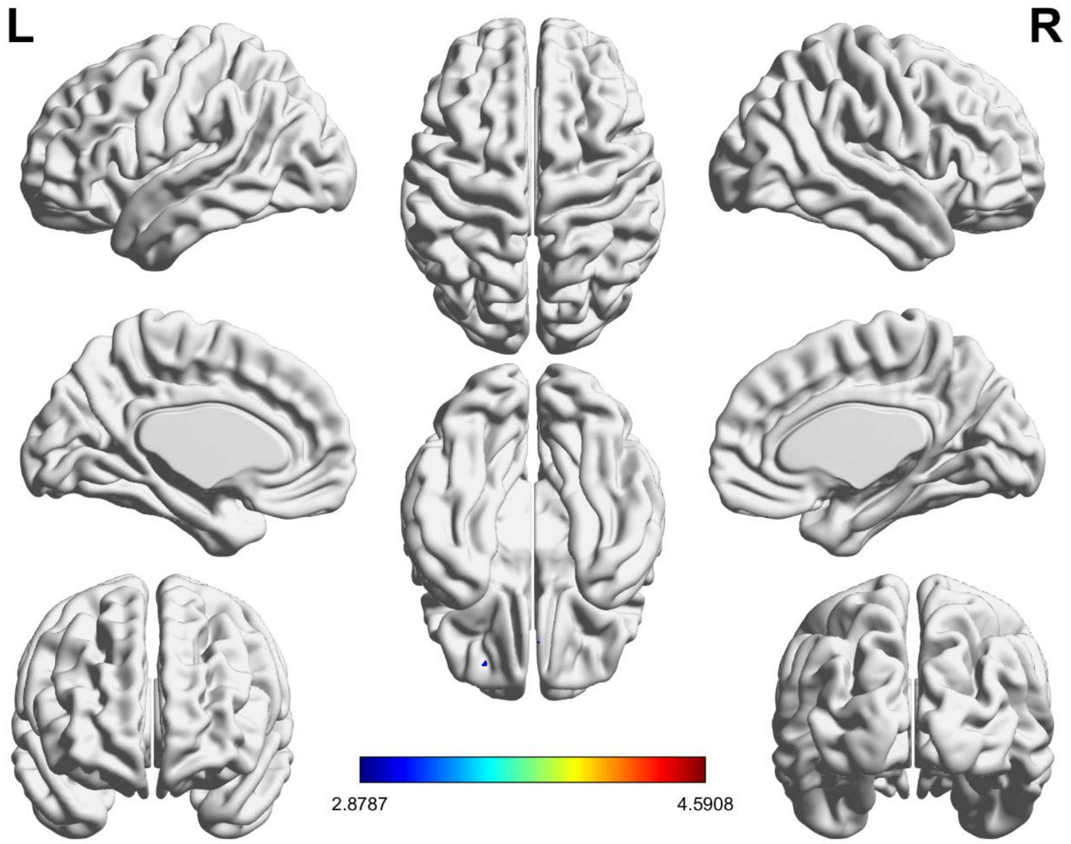

Figure S2.

Group comparisons of static fALFF between the right side SSHL and HC groups.

(Gaussian random field correction; voxel-level $p < 0.001$, cluster-level $p < 0.01$). SSHL, sudden sensorineural hearing loss; HC, healthy controls; L, left; R, righ.

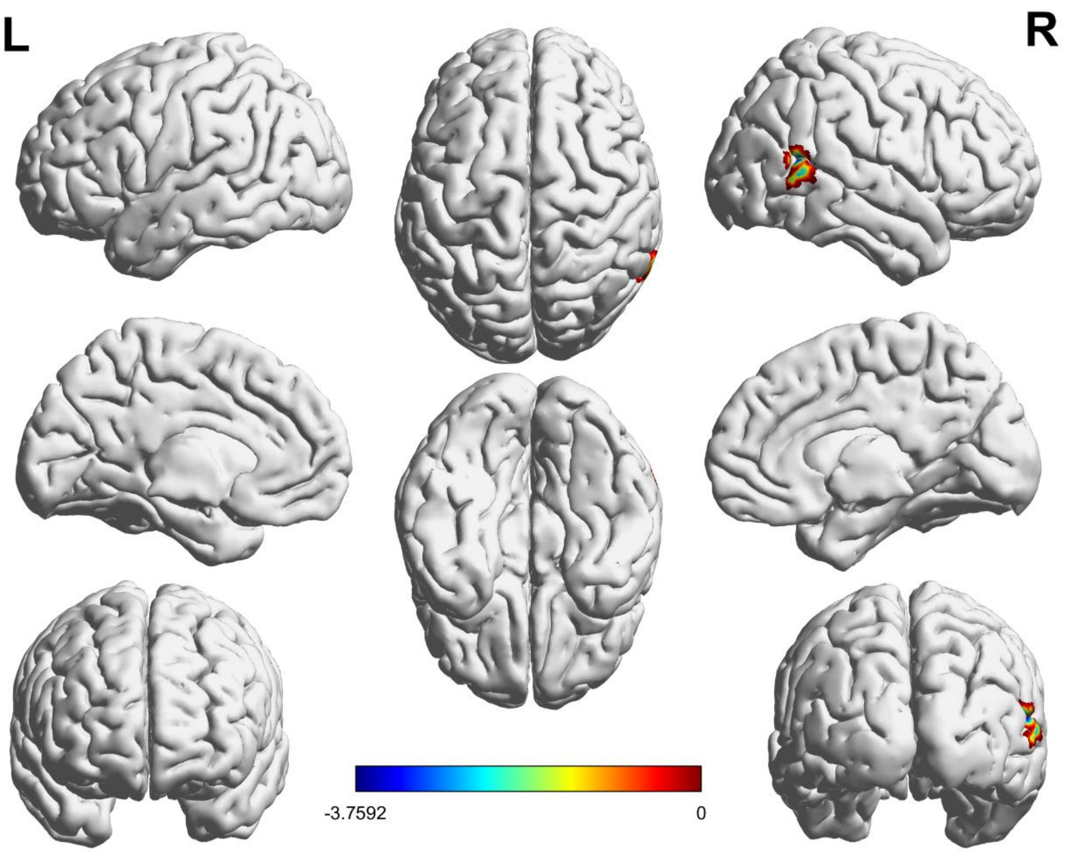

Figure S3.

Group comparisons of static fALFF between the left side SSHL and right side SSHL groups.

(Gaussian random field correction; voxel-level $p < 0.001$, cluster-level $p < 0.01$). SSHL, sudden sensorineural hearing loss; HC, healthy controls; L, left; R, righ.

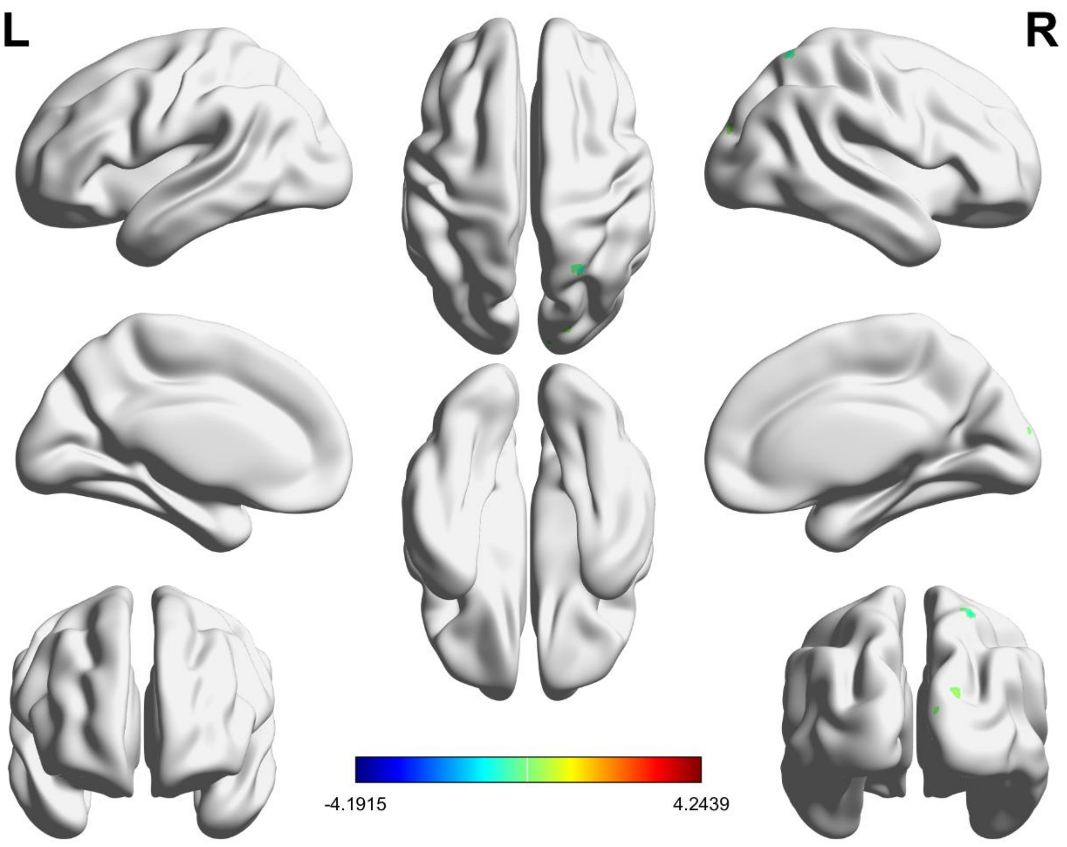

Figure S4.

Group comparisons of dynamic fALFF between the left side SSHL and HC groups

(Gaussian random field correction; voxel-level p < 0.001, cluster-level p < 0.01). SSHL, sudden sensorineural hearing loss; HC, healthy controls; L, left; R, righ.

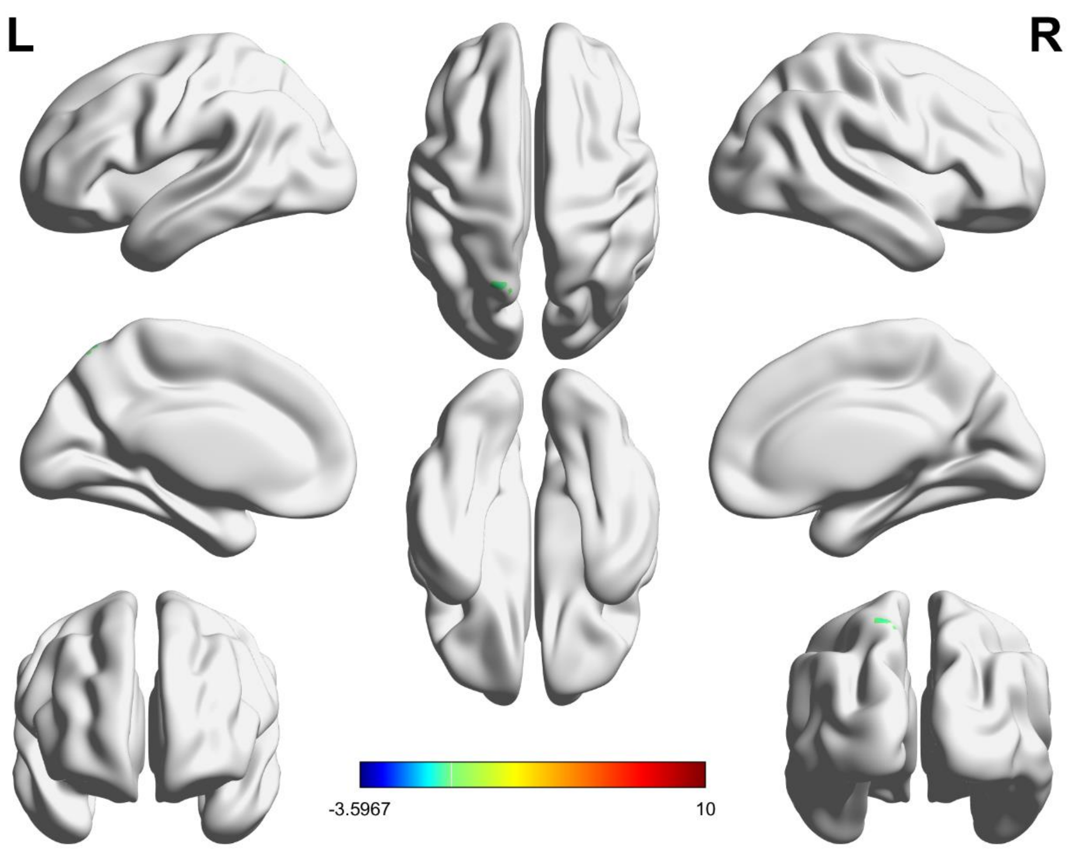

Figure S5.

Group comparisons of dynamic fALFF between the left side SSHL and right side SSHL groups.

(Gaussian random field correction; voxel-level $p < 0.001$, cluster-level $p < 0.01$). SSHL, sudden sensorineural hearing loss; HC, healthy controls; L, left; R, righ.
